# Circulating levels of PD-L1 and Galectin-9 are associated with patient survival in surgically treated Hepatocellular Carcinoma independent of their intra-tumoral expression levels

**DOI:** 10.1038/s41598-019-47235-z

**Published:** 2019-07-23

**Authors:** Kostandinos Sideras, Robert A. de Man, Susan M. Harrington, Wojciech G. Polak, Guoying Zhou, Hannah M. Schutz, Alexander Pedroza-Gonzalez, Katharina Biermann, Shanta Mancham, Bettina E. Hansen, R. Bart Takkenberg, Anneke J. van Vuuren, Qiuwei Pan, Jan N. M. Ijzermans, Stefan Sleijfer, Dave Sprengers, Haidong Dong, Jaap Kwekkeboom, Marco J. Bruno

**Affiliations:** 1 Erasmus MC-University Medical Center, Department of Gastroenterology and Hepatology, Rotterdam, The Netherlands; 2 Mayo Clinic College of Medicine, Department of Immunology, Rochester, MN USA; 3 Erasmus MC-University Medical Center, Department of Surgery, Rotterdam, The Netherlands; 4 Erasmus MC-University Medical Center, Department of Pathology, Rotterdam, The Netherlands; 5 Academic Medical Center, Tytgat Institute for Liver and Intestinal Research, University of Amsterdam, Amsterdam, The Netherlands; 6 Erasmus MC-University Medical Center, Erasmus MC Cancer Institute, Department of Medical Oncology, Rotterdam, The Netherlands; 7 Present Address: Laboratory of Immunology Research, Medicine, Faculty of Higher Studies Iztacala, National Autonomous University of Mexico, FES-Iztacala, UNAM, Mexico City, Mexico

**Keywords:** Cancer microenvironment, Pancreatic cancer, Translational immunology

## Abstract

Tumor expression of immune co-inhibitory ligands, such as PD-L1 and Galectin-9, have potential prognostic value in Hepatocellular Carcinoma (HCC). Circulating levels of these molecules, however, have hardly been studied. This study aims to assess the prognostic significance of circulating PD-L1 and circulating Galectin-9 in patients with resected HCC, and to compare their prognostic significance to the intra-tumoral expression of these same molecules. Archived tissues and stored peripheral blood samples from 81 patients who underwent HCC resection or liver transplantation, with curative intent, were used. Immunohistochemistry was performed to determine intra-tumoral expression of PD-L1 and Galectin-9, while ELISA was used to quantify their respective circulating levels. High circulating PD-L1 (HR 0.12, 95%CI 0.16–0.86, p = 0.011) and high circulating Galectin-9 (HR 0.11, 95%CI 0.15–0.85, p = 0.010) levels were both associated with improved HCC-specific survival. Surprisingly, there was no correlation between circulating levels of PD-L1 and Galectin-9 and their intra-tumoral expression levels. In fact, circulating levels of PD-L1 and Galectin-9 were predictive of HCC-specific survival independently of intra-tumoral levels and baseline clinicopathologic characteristics. Combined analysis of circulating levels and intra-tumoral expression of PD-L1 (HR 0.33, 95%CI 0.16–0.68, p = 0.002) and Galectin-9 (HR 0.27, 95%CI 0.13–0.57, p = 0.001) resulted in more confident prediction of survival. In conclusion, circulating PD-L1 and Galectin-9 levels prognostically differentiate resected HCC patients, independently of their intra-tumoral expression. Combining circulating and intra-tumoral expression levels of PD-L1 or Galectin-9 further improves the prognostic values of these immune biomarkers.

## Introduction

Worldwide over half a million people die from HCC^1^. Only 20% of patients with HCC are diagnosed early enough to be candidates for curative treatments such as resection, local ablation or liver transplantation^2^. Once advanced disease is diagnosed, HCC is incurable and overall survival can be only modestly extended with sorafenib^3^.

Developments in our understanding of tumor immunology^4^ have brought forth new therapeutic strategies against cancer^5^. Currently, the most successful immunotherapeutic strategies are those that are designed to overcome immune resistance mechanisms by using antibodies that abrogate co-inhibitory receptor-ligand interactions, the so-called negative immune checkpoint inhibitors^6^. Recent approval of anti-CTLA-4 and anti-PD-1 antibodies for the treatment of advanced melanoma, non-small cell lung cancer, renal cell carcinoma and bladder cancer has made it clear that immunotherapy is the new wave of anti-cancer treatments. Immunotherapy clinical trials are now ongoing in many cancers, including HCC^7^, and it is likely that these therapies will be approved in the future in other cancer types as well. In view of the high costs and occasional severe toxicity of these novel therapies, immune specific biomarkers that can predict which patients will benefit are urgently needed. Two such recent promising immune biomarkers in HCC are PD-L1 and Galectin-9 (Gal-9).

PD-L1 is a ligand that binds PD-1, a co-inhibitory receptor expressed on activated T cells. Binding of the ligand PD-L1 to its receptor PD-1 transduces a negative signal into T-cells inhibiting their activation. In HCC the PD-L1/PD-1 interaction impairs effector T-cell function and *in vitro* disruption of this interaction restores the function of tumor-derived effector T-cells^8^. PD-L1 is known to be expressed by HCC cells^9–13^. We have previously shown that tumor PD-L1 protein expression is a promising prognostic biomarker in HCC^14^. In addition, tumor PD-L1 protein expression has shown promise as a predictive biomarker to identify cancer patients that respond to anti-PD1 immunotherapy^15,16^.

Gal-9 is a glycan-binding protein and an important modulator of T-cell function^17^. Gal-9 causes T-cell inhibition and apoptosis through its binding to the co-inhibitory receptor TIM-3 and blockade of the interaction between Gal-9 and TIM-3 reinvigorates *ex vivo* responses of T-cells of HCC and melanoma patients to tumor antigens^8,18^. Humanized antagonistic antibodies against TIM-3 are currently in preclinical development^19^. In addition, binding of Gal-9 to CD44 enhances the differentiation of immunosuppressive T regulatory cells^20^. A direct anti-metastatic role for Gal-9 has also been described^21,22^. We and others have demonstrated that Gal-9 is also expressed by HCC tumor cells and that Gal-9 protein expression is a potential prognostic biomarker in HCC^14,23,24^.

However, in addition to cell-bound expression, soluble forms of PD-L1 and Gal-9 exist in the circulation. These circulating forms of PD-L1 and Gal-9 have been poorly studied in cancer patients. Circulating levels of PD-L1 have been examined in renal-cell cancer^25^, lung cancer^26^, gastric cancer^27^, pancreatic cancer^28^ and HCC^29^. Elevated levels of circulating Gal-9 have been observed in metastatic colon cancer^30^ and in benign inflammatory liver diseases such as chronic hepatitis-C and active chronic hepatitis-B infection^31,32^. However, no study has investigated the relationship of circulating Gal-9 to cancer survival before.

One may hypothesize that circulating forms of PD-L1 or Gal-9 would correlate with their tumor tissue expression status, since release from tumor cells, or from the tumor microenvironment, may be the source of these molecules in the circulation. In that case, circulating PD-L1 and Gal-9 may act as preferred biomarkers given the easier accessibility of peripheral blood when compared to tumor tissue. On the other hand, these physiologic molecules may have functions in the circulation that are independent of the immune interactions ongoing in the tumor microenvironment. Thus, the potential for the circulating levels of PD-L1 and Gal-9 to act as independent prognostic biomarkers, or independent predictors of immunotherapy treatment efficacy, exists. Thus, the aims of our study were to examine how circulating levels of PD-L1 and Gal-9 compare to tissue expression of these molecules and whether circulating levels have the potential to replace tissue expression, or add to tissue expression, as potential immune biomarkers in HCC patients undergoing surgical resection.

## Materials and Methods

### Patient population and tissue samples

Archival blood samples (59 serum samples and 22 plasma samples) and formalin fixed paraffin-embedded tissue samples from 81 patients who underwent hepatic resection or liver transplantation for HCC at Erasmus MC-University Medical Center between January 2007 and March 2013, were used for this study. All patients had undergone procedures with curative intent.

### Ethical approval

Medical ethical approval for this study was obtained from the Medical Ethics Committee of Erasmus MC (MEC-2015-237). All patients gave informed consent for the use of their blood and tissue in medical research. All procedures performed in studies involving human participants were in accordance with the ethical standards of the institutional and/or national research committee and with the 1964 Helsinki declaration and its later amendments or comparable ethical standards.

### Enzyme-linked immunosorbent assay

Sera or plasma samples were drawn pre-operatively (median 1 day, mean 35 days) and were stored at −80 °C. Soluble PD-L1 was measured using a validated ELISA^25,33^ in the laboratory of Dr. Haidong Dong (Mayo Clinic, MN., US). The dynamic range of this ELISA is: 86–3670 pg/ml. Limit of detection: 84 pg/ml. Recovery from heparin plasma: 84–105%. Soluble Gal-9 was performed using an ELISA kit according to the manufacturer’s protocol (Uscn Life Science Inco, Wuhan China). This ELISA kit has been used in several published studies^32,34,35^. The dynamic range of this ELISA is 7.8–500 pg/ml. Limit of detection 3.3 pg/ml. Recovery from serum: 91–104%. Recovery from heparin plasma: 87–102%. All samples were tested in duplicate and mean values were used for analysis.

### Tissue microarray (TMA) construction

TMAs were constructed as previously described^14^. In brief, three 0.6 mm cores were taken from the tumorous areas and two 0.6 mm cores were taken from the surrounding tumor free liver tissue of each tissue block. The tumorous areas with vital tissue were marked by an experienced pathologist using archived H&E glass slides. The TMAs were made using an automated tissue-arrayer ATA-27 (Beecher Instruments, Silver Springs MD, USA).

### Immunohistochemistry and scoring

Immunohistochemistry and scoring was performed as previously described^14^. In brief 4 µm thick sections were mounted on Superfrost Plus^TM^ slides. The sections were deparaffinized and rehydrated. Endogenous peroxidase activity was blocked with 0.3% H_2_O_2_ for 15 minutes. Antigen retrieval was performed in a microwave for 10 minutes using the appropriate antigen retrieval buffer. After serum block, primary antibodies were applied at 4 °C overnight. The primary antibodies were PD-L1 clone 405.9A11^36^ (kindly provided by Dr. Gordon J. Freeman, Dana-Farber Cancer Institute, Boston, MA, USA), and Gal-9 goat polyclonal^31^ (R&D systems). HRP-conjugated anti-mouse or anti-goat IgG polymer secondary antibody (Envision^TM^, DAKO) was then applied for 1 hour, followed by diaminobenzadine (DAB) as the chromogen detection method. The slides were stained with haematoxylin followed by dehydration. Negative controls consisted of omission of the primary antibody and appropriate positive control tissues included in the TMAs were used to evaluate specificity of all antibodies.

Scoring was performed by 2 independent investigators blinded to clinical outcome and differences resolved by mutual agreement. Only cytoplasmatic staining was observed for both antibodies. Intensity of tumor cell and hepatocyte staining was scored in a scale from zero to three. Intra-core heterogeneity of staining intensity of tumor cells or hepatocytes was rarely observed, thus percentages of positive tumor cells were not analyzed. Average values of the scores of the different cores were used for analysis. Patients with any evaluable PD-L1 or Gal-9 staining on their tumor cells were considered to have high expression, while patients with complete absence of staining were considered to have low expression.

### Statistical analysis

All analyses were performed in duplicate. Survival curves were estimated by the Kaplan-Meier method. Survival was calculated from the date of surgery to the date of event (recurrence or death), or date of last follow up. The patients who died from causes other than HCC were censored at their time of death. The log-rank test was used to assess differences between survival curves of different groups, while for biomarkers with three linearly associated levels the linear trend for factor levels was used. Optimal high vs low values were established by examining a grid of cutoffs and choosing the cutoff with the lowest −2 log likelihood. For multivariate analysis, the Cox proportional Hazard regression analysis was used. The associations between clinicopathologic parameters with immune biomarkers were examined using the χ^2^ tests or the T-test as appropriate. For the correlations between the circulating and intra-tumoral immune biomarkers the Spearman Correlation test was performed. Sensitivity analysis was performed in relation to sample source (serums versus plasma), type of surgery (resection versus liver transplantation) and the use of the TNM classification system. The statistical analysis was performed using the SPSS© 21 software.

## Results

### Patients and clinicopathologic characteristics

Pre-operative circulating PD-L1 and Gal-9 levels were studied in 81 HCC patients who underwent hepatic resection or liver transplantation. Plot diagrams of circulating PD-L1 and Gal-9 levels can be seen in Supplementary Fig. 1. Median time to cancer recurrence was 29.7 months and median HCC-specific survival was 34.2 months. Baseline clinicopathologic characteristics can be found in Table 1 (etiology of liver disease in Supplementary Table 1). In univariate analysis, tumor size >3 cm (HR 3.0, 95%CI 1.1–8.2, p = 0.032) predicted HCC-specific mortality and a pre-operative α-fetoprotein (AFP) level >100 µgl^−1^ (HR 2.1, 95%CI 0.9–5.3, p = 0.092) showed a non-significant trend toward predicting HCC-specific mortality. Number of lesions, tumor differentiation, vascular invasion and cirrhosis did not predict HCC-specific mortality in our cohort. Tumor size >3 cm also predicted HCC recurrence (HR 2.5, 95%CI 1.2–5.3, p = 0.012). Circulating PD-L1 and Gal-9 were not associated with any of the above clinicopathologic characteristics with the exception of a positive association between circulating PD-L1 (but not Gal-9) and cirrhosis (p = 0.045).

**Table 1:** .

Baseline characteristics		N or median (% or range)
Age		60 (23–79)
Gender (male/female)		58 (71.6)/23 (28.4)
Type of Surgery	Resection	58 (71.6)
	Liver Transplantation	23 (28.4)
AFP before resection		7.5 ug/l (1–15,000)
Cirrhosis		44 (54.3)
Viral hepatitis	Hepatitis-B^a^	17 (21.0)
	Hepatitis-C^b^	10 (12.3)
Tumor size (cm)		3.6 (1–25)
Number of lesions	Single	59 (72.8)
	Multiple	22 (27.2)
Vascular invasion		46 (64.8)
Tumor differentiation	Well	26 (32.1)
	Moderate	45 (55.6)
	Poor	10 (12.3)
TNM stage	I	22 (27.5)
	II	53 (66.3)
	IIIA	5 (6.3)
HCC recurrence		36 (44.4)
HCC specific death		21 (25.9)

^a^HBsAg(+) and/or anti-HBc positive.

^b^Anti-HCV positive. Baseline characteristics.

### Association of pre-operative circulating PD-L1 and Gal-9 with recurrence and survival

**Figure 1:**
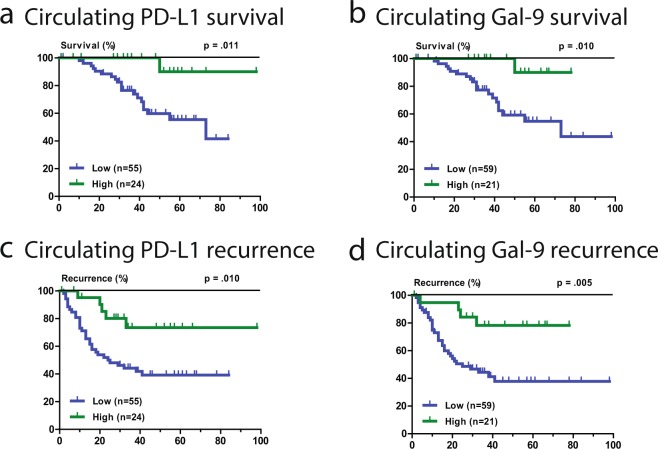
Pre-operative circulating PD-L1 and Galectin-9 levels are associated with HCC-specific survival and tumor recurrence. Kaplan-Meier graphs showing: (**a**) HCC-specific mortality in HCC patients with high or low circulating PD-L1 concentrations. (**b**) HCC-specific mortality in HCC patients with high or low circulating Gal-9 concentrations. (**c**) Recurrence-free survival in HCC patients with high or low circulating PD-L1 concentrations. (**d**) Recurrence-free survival in HCC patients with high or low circulating Gal-9 concentrations.

Median pre-operative circulating PD-L1 concentration was 383 pg/ml (intra-quartile range (IQR) 206–774 pg/ml), and median pre-operative circulating Gal-9 concentration was 21 pg/ml (IQR 3–44 pg/ml). With an optimal cutoff of 700 pg/ml, high circulating PD-L1 was associated with improved HCC-specific survival (HR 0.12, 95%CI 0.16–0.86, p = 0.011), and with a cutoff of 42 pg/ml high circulating Gal-9 was also associated with improved HCC-specific survival (HR 0.11, 95%CI 0.15–0.85, p = 0.010). The respected Kaplan-Meier curves are shown in Fig. 1a,b. Using the same cutoffs, similar significant relationships between circulating PD-L1 and Gal-9 and HCC recurrence were found (Fig. 1c,d). There was no correlation between circulating PD-L1 and Gal-9 levels (Spearman Correlation = 0.101, p = 0.38). In multivariate analysis, together with clinicopathologic characteristics, pre-operative circulating Gal-9 (p = 0.022) could independently predict HCC-specific survival, while pre-operative circulating PD-L1 (p = 0.077) and AFP > 100 µgl^−1^ (p = 0.060) showed non-significant trends to association with HCC-specific survival.

### Sensitivity analysis

In the majority of patients PD-L1 and Gal-9 were measured in sera, but from 22 patients only plasma samples were available. When the patients with plasma samples were excluded from the analysis, the Kaplan-Meier survival curves for the remaining 59 patients are similar to Fig. 1a,b (see Supplementary Fig. 2a,b for comparison). In addition, the Kaplan-Meier survival curves of the 22 patients with plasma samples follow the same general direction as the patients with serum samples (Supplementary Fig. 2c,d). Similarly, when the cohort is split between patients who underwent hepatic tumor resection versus liver transplantation, the Kaplan-Meier survival curves of circulating PD-L1 and circulating Gal-9 levels look similar and follow the same direction (Supplementary Fig. 3a–d). Thus, our results do not depend on the use of serum versus plasma samples or the type of surgery performed. Finally, sensitivity analysis was performed by using the TNM stage instead of the baseline clinicopathologic parameters of tumor size and number of lesions. The inclusion of the TNM staging in the multivariate analysis produced nearly identical results (data not shown).

### Association of intra-tumoral PD-L1 and Gal-9 with recurrence and survival

**Figure 2:**
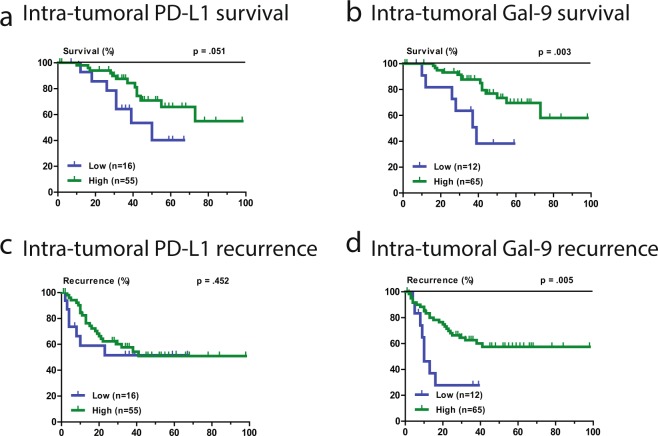
Intra-tumoral PD-L1 and Galectin-9 expressions are associated with HCC-specific survival. Kaplan-Meier graphs showing: (**a**) HCC-specific mortality in patients with high or low intra-tumoral PD-L1 staining. (**b**) HCC-specific mortality in patients with high or low intra-tumoral Gal-9 staining. (**c**) Recurrence-free survival in patients with high or low intra-tumoral PD-L1 staining. (**d**) Recurrence-free survival in patients with high or low intra-tumoral Gal-9 staining. Please note that the data on the intra-tumoral expression of PD-L1 and Gal-9 and patient survival, of 58 out of the 81 patients in this figure, have been included in a previous publication^14^.

The intra-tumoral expression of PD-L1 and Gal-9, in combination with other immune inhibitory molecules, has been studied by our group before, in an overlapping cohort^14^. Specifically, 58 patients from the previous cohort of 154 resected HCC patients had available stored peripheral blood, and were thus included in the current study. An additional 23 patients, unique in the present study, had available stored peripheral blood and thus had their corresponding tumor tissue immunohistochemically stained for PD-L1 and Gal-9. Examples of PD-L1 and Gal-9 immunohistochemical staining of tumor tissues are shown in Supplementary Fig. 4. In the current study PD-L1 expression in tumor cells was seen in 78% of patients and Gal-9 in 84% of patients. Patients with any evaluable PD-L1 or Gal-9 staining on their tumor cells were considered to have high expression, while patients with complete absence of staining were considered to have low expression. Patients with high intra-tumoral PD-L1 (HR 0.41, 95%CI 0.16–1.04, p = 0.051) and Gal-9 (HR 0.26, 95%CI 0.10–0.68, p = 0.003) had an improved HCC-specific survival (Fig. 2a,b), in agreement with our previous observations^14^. The respective relationships of intra-tumoral PD-L1 and Gal-9 with HCC recurrence can be seen in Fig. 2c,d. In multivariate analysis, intra-tumoral Gal-9 (p = 0.004), intra-tumoral PD-L1 (p = 0.035) and AFP > 100 µgl^−1^ (p = 0.004) were independent predictors of HCC-specific survival.

### Circulating versus intra-tumoral and tumor-free liver PD-L1 and Gal-9

Next we examined if pre-operative circulating levels of PD-L1 and Gal-9 reflected intra-tumoral expression. There was no correlation between intra-tumoral PD-L1 and circulating PD-L1 (Spearman Correlation = 0.135, p = 0.27) or between intra-tumoral Gal-9 and circulating Gal-9 (Spearman Correlation = 0.092, p = 0.43). Box-and-whisker plot graphs supporting the above lack of association can be seen in Supplementary Fig. 5. In multivariate analysis, both intra-tumoral Gal-9 (p = 0.003) and circulating Gal-9 (p = 0.031) independently predicted HCC-specific survival, together with the presence of cirrhosis (p = 0.036) and AFP > 100 µgl^−1^ (p = 0.050). In the case of PD-L1, both intra-tumoral PD-L1 (p = 0.056) and circulating PD-L1 (p = 0.067) showed a non-significant trend towards independently predicting HCC-specific survival, together with AFP > 100 µgl^−1^ (p = 0.039). In addition, there was no correlation between circulating levels and expression levels of PD-L1 or Gal-9 on hepatocytes in the surrounding tumor free liver tissue.

### Combining circulating and intra-tumoral expression improves prognostication

**Figure 3:**
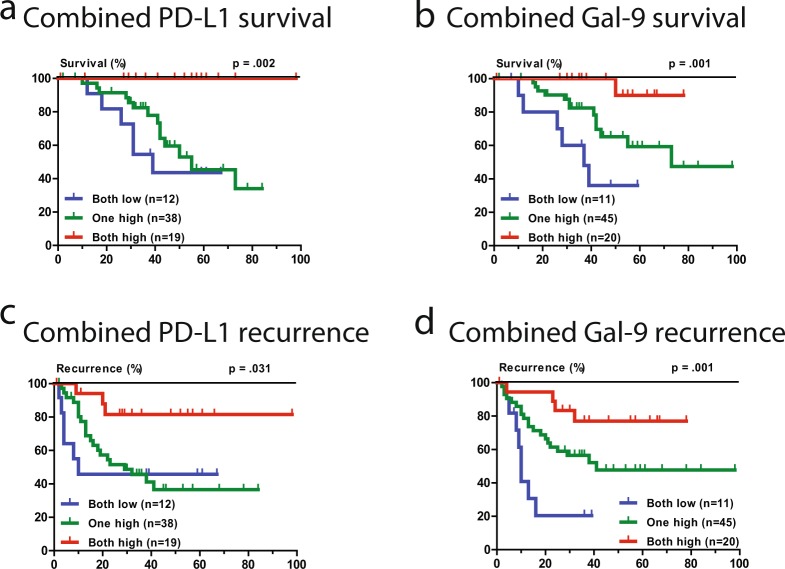
Kaplan-Meier graphs of combined pre-operative circulating and intra-tumoral PD-L1 and Galectin-9. (**a**) HCC-specific mortality of combined circulating and intra-tumoral PD-L1. (**b**) HCC-specific mortality of combined circulating and intra-tumoral Gal-9. (**c**) Recurrence-free survival of combined circulating and intra-tumoral PD-L1. (**d**) Recurrence-free survival of combined circulating and intra-tumoral Gal-9.

Given the lack of association between intra-tumoral expression and circulating levels of the respective ligands, and their independence for predicting survival in multivariate analysis, we examined if combining both improved the prognostication of patients with resected HCC. Figure 3a,b show the Kaplan-Meier survival curves of the combined (intra-tumoral and circulating) PD-L1 and Gal-9 biomarkers in relation to HCC specific survival, respectively. Interestingly, patients with both high levels of circulating and intra-tumoral PD-L1 showed 100% survival (HR 0.33, 95%CI 0.16–0.68, p = 0.002). In addition, combined analysis of circulating and intra-tumoral Gal-9 could distinguish 3 groups of patients with distinct survival curves (HR 0.27, 95%CI 0.13–0.57, p = 0.001). Thus, combining circulating and intra-tumoral expression levels further improves the prognostic values of these immune biomarkers. Figure 3c,d show similar relationships of combined analysis of circulating and intra-tumoral PD-L1 and Gal-9 with HCC recurrence. In multivariate analysis both the PD-L1 combined biomarker and the Gal-9 combined biomarker, together with AFP > 100 µgl^−1^ and cirrhosis, are independent predictors of HCC-specific survival (Table 2). In sensitivity analysis when the TNM staging is used nearly identical results were produced (Supplementary Table 2).

**Table 2:** Multivariate Cox proportional Hazard regression analysis of patients’ survival.

Variables	HR	95% CI	p-value
Multivariate Cox proportional Hazard regression analysis of patients’ survival			
Size >3 cm	2.58	0.74–8.95	0.136
Number of lesions	0.47	0.13–1.75	0.260
Tumor differentiation	0.97	0.40–2.38	0.953
Cirrhosis	3.25	1.10–9.60	**0.033**
AFP > 100 µgl^−1^	4.60	1.32–16.0	**0.017**
Combined PD-L1	0.38	0.17–0.88	**0.023**
Combined Gal-9	0.16	0.05–0.47	**0.001**

## Discussion

We show that both pre-operative circulating PD-L1 and Gal-9 are able to differentiate resected HCC patients prognostically. High levels of circulating PD-L1 and Gal-9 are associated with delayed recurrence and better survival of patients undergoing curative intent surgery for HCC. Interestingly, circulating levels of PD-L1 and Gal-9 were not correlated to intra-tumoral expression, and showed prognostic value independently of intra-tumoral expression. We also show that when intra-tumoral expression and circulating levels of PD-L1 and Gal-9 are combined prognostication improves even further.

No study has yet examined the prognostic significance of circulating Gal-9 in any cancer. On the other hand, the prognostic role of circulating PD-L1 has been examined in a few cancer types before^25–27,37^, including a recent study in HCC by Finkelmeier *et al*.^29^. Finkelmeier *et al*., examined the prognostic significance of circulating PD-L1 in 219 patients with HCC and, in contrast to our study, found that higher levels of circulating PD-L1 were associated with worse patient survival. It is important to note some differences between the two studies. Our study included only patients who were treated by tumor resection (n = 58) or liver transplantation (n = 23) with curative intent, while Finkelmeier *et al*., included patients from all stages of HCC of which the majority were treated by local ablation or palliative therapy with sorafenib. Only 21% of their patients were treated with resection (n = 26) or liver transplantation (n = 19). It is entirely possible that circulating PD-L1 may have different roles in a setting of a small amount of resectable disease versus an advanced palliative setting with a large tumor burden. In the early curative setting, high levels of circulating PD-L1 may represent adaptive immune resistance of the tumor against successful immune attack, resulting in increased expression and release of PD-L1 by cells in the tumor in response to pro-inflammatory cytokines secreted by infiltrating immune cells. In this setting high levels of circulating PD-L1 would be associated with good prognosis. In the advanced metastatic setting the anti-tumor immune system may be overburdened and no longer able to suppress tumor growth. In this setting high levels circulating PD-L1 may reflect increased tumor load and therefore be associated with worse prognosis. Indeed, in the Finkelmeier study circulating PD-L1 levels were positively associated with more advanced disease, while this was not the case in our cohort (no association with TNM stage, number of lesions, tumor size). Similarly, in advanced renal cell carcinoma and advanced lung cancer high circulating PD-L1 levels were positively correlated with disease stage and with worse prognosis^25,26^. On the other hand, no correlation between circulating PD-L1 levels and outcome was found in advanced pancreatic cancer^28^. Such contrasting observations may not be surprising since such differences have been similarly observed in the setting of intra-tumoral PD-L1 expression where the prognostic significance of PD-L1 differs depending on the cancer type and stage. An additional difference with the Finkelmeier *et al*. study is the fact that in our study 54% of patients did not have cirrhosis and 25% of patients had no identifiable underlying liver disease. In contrast, in the Finkelmeier *et al*. study, all patients had an identifiable underlying liver disease and all patients were assigned a Child-Pugh score, indicating that all patients had cirrhosis. Thus, our opposite results may also be due to the inclusion of many HCC patients without underlying liver disease in our study. Finally, the use of different ELISA assays could be another explanation for the differing results although, despite using different assays, both studies observed similar median circulating PD-L1 levels (383 vs 500 pg/ml in our study and in the Finkelmeier *et al*. study, respectively) and used similar cutoff values (700 vs 800 pg/ml).

An additional consideration that may influence the prognostic role of PD-L1 has to do with the source and the particular form of the circulating ligand. One hypothesis is that PD-L1can be actively shed from membrane-bound PD-L1 expressing tumor cells and/or hepatocytes. Soluble PD-L1 has indeed been shown to be released into cell culture supernatants by several, but not all, membrane PD-L1 expressing tumor cell lines^25^, suggesting that expression of soluble and membrane-bound PD-L1 are differentially regulated. Preliminary data have shown involvement of matrix metalloproteinases in shedding of membrane-bound PD-L1^38^. More recently it has been shown that tumor cells can produce soluble PD-L1 by alternative splicing^39^. Moreover, PD-L1 can be released from tumor associated macrophages and dendritic cells. It has been shown by us and by other research groups that tumor associated macrophages and dendritic cells express PD-L1 in the tumor microenvironment in HCC^8,12,13,40^. Myeloid cells, especially mature dendritic cells, have been shown to release soluble PD-L1 *in vitro*^39,41^ and it has been recently shown that circulating activated macrophages in ovarian cancer patients also express high levels of PD-L1^42,43^. Finally, another possible source of soluble PD-L may be circulating tumor cells, which have been shown to express PD-L1 in breast cancer patients^44^. Whatever the source of circulating PD-L1 may be, the circulating ligand is known to retain its PD-1 binding domain and immunosuppressive properties^25,39^. Regarding the form of soluble PD-L1, recent studies have shown that PD-L1 can be released from tumor cells in the form of exosomes. Interestingly, pre-treatment levels of circulating exosomal PD-L1 were higher in melanoma patients that failed to respond to pembrolizumab therapy than in non-responders, but this difference was not observed for total circulating PD-L1^45^. In head and neck cancer, levels of PD-L1 carried by exosomes correlated with disease activity but total plasma PD-L1 levels did not^46^. Therefore, the form of circulating PD-L1, either expressed in circulating exosomes or in non-bound form, may additionally explain why high circulating PD-L1 levels are associated with worse or better prognosis depending on the cancer setting. Another theoretical possibility for differences between studies is the use of differing ELISA systems. It is important to note however that Frigola *et al*.^25^, found that high circulating PD-L1 is associated with worse patient survival in Renal Cell Carcinoma, results opposite to ours, using the same ELISA and the same laboratory where our assays were performed. Thus, it is likely that biologic factors, rather than technical factors, account for the differences in results between our study and that of Frigola *et al*., as we discuss above.

Like PD-L1, Gal-9 is not only expressed on tumor cells, but also at high levels on tumor-associated macrophages in HCC^8,23^. Gal-9 does not have a signal peptide, and its secretion must involve a non-classical pathway. Cleavage from the cell surface by matrix-metalloproteinases has been suggested as a secretion pathway^47^, while other studies showed that cancer cells can secrete Gal-9 via exosomes^48^. Therefore, circulating levels of these molecules are probably not passive reflections of tissue expression. In addition, while their expression in tumor tissues probably determines their local effects, their circulating counterparts may exert systemic effects, e.g. by inhibiting systemic immunity or, in case of Gal-9, prevent formation of distant metastasis by hampering extravasation of tumor cells into other tissues^21,22^. This means that future studies on the role of PD-L1 and Gal-9 as prognostic or predictive markers should take both intra-tumoral and circulating levels into consideration to maximize the potential for biomarker optimization.

The current study focuses on circulating, pre-operative, PD-L1 and Gal-9 levels and the interplay of these circulating levels to the intra-tumoral expression of PD-L1 and Gal-9. In contrast, the isolated intra-tumoral protein expression of PD-L1 and Gal-9 in HCC has been examined in several studies before^9–13,23,24^. We have previously studied the prognostic value of intra-tumoral expression of PD-L1 and Gal-9^14^. Our current results regarding intra-tumoral expression of PD-L1 and Gal-9 (Fig. 2) are in agreement with our previous observations. This agreement was expected given the significant overlap in patients between the two studies since 58 of the 81 patients were included in the previous study. It was necessary, however, to redo the analysis in the new cohort as this was the only way to compare circulating with intra-tumoral levels. Nevertheless, it is important to note that despite the significantly smaller size of the current cohort, and the addition of new patients, we show, again, that high intra-tumoral expression of PD-L1 and Gal-9 are still associated with improved survival. The paradoxical observation that high intra-tumoral levels of immune inhibitory molecules are related to better, rather than worse, survival has been noted before^49,50^ and it is attributed to the phenomenon of adaptive immune resistance. Specifically, it has been observed that immune inhibitory molecules can be overexpressed on tumor cells and hepatocytes in response to IFN-γ or lymphocytic infiltration^49,51–53^.

Our original hypothesis was that circulating PD-L1 and Gal-9 would represent molecules that are passively released from tumor cells and therefore would reflect intra-tumoral expression of these molecules. However, similar to recent PD-L1 studies in B-cell lymphoma and pancreas cancer^28,37^, we found that circulating levels of both PD-L1 and Gal-9 were not correlated to intra-tumoral expression. In addition, circulating PD-L1 and Gal-9 levels, in the current study, did not correlate with their expression on hepatocytes in tumor-free liver tissues. Instead, we found that circulating levels and intra-tumoral expression contributed independently to prognostication. It is thus possible that intra-tumoral and circulating forms of PD-L1 and Gal-9 have different pathophysiologic origins and/or functions.

We found an association between circulating PD-L1 and the presence of cirrhosis. A similar relationship was suggested by Finkelmeier *et al*. who noted that circulating PD-L1 levels was associated with the severity of cirrhosis^29^. On the other hand no association was found between circulating Gal-9 and cirrhosis. While this observation appears to contradict a recent observation made by Fujita *et al*.^54^, where a positive association was reported between circulating Gal-9 and cirrhosis, it is likely that differences in cohort characteristics are likely to explain the discrepancy. Only about half the patients in the Fujita et. al., study had HCC while about half the patients had chronic hepatitis-C, a much larger proportion than in our study. It should be noted, however, that while not statistically significant, a higher mean circulating Gal-9 level was indeed observed in patients with cirrhosis in our study (36.3 pg/ml vs 25.5 pg/ml) indicating that sample size may be another explanation for the difference, and that perhaps our results are not that discrepant with the results of the Fujita *et al*., study. In addition, it is important to keep in mind that in multivariate analysis the association of circulating PD-L1 and Gal-9 with survival was independent of the presence or absence of cirrhosis. Thus cirrhosis was not a confounder of PD-L1 or Gal-9 levels in regards to patient survival.

Our study has several strengths. It is the first study to examine the role of circulating Gal-9 in HCC and one of only two studies, to date, to examine the role of circulating PD-L1 in HCC. In addition, few studies have studied circulating levels of these molecules in other cancers, thus this is a fairly unexplored field of research. Most importantly both the intra-tumoral expression and the circulating levels of these molecules were compared. The observation that intra-tumoral and circulating levels independently contribute to prognostication, is novel. Testing for the expression of these molecules in the surrounding tumor free liver tissue also allowed us to show that it is not the expression on hepatocytes in the diseased liver that determines circulating levels of PD-L1 and Gal-9. Our study has also limitations. One limitation of our study is the use of both serum and plasma samples for our analysis. While we show in Supplementary Fig. 2 that the use of serum versus plasma samples does not influence our main results, serum samples should be probably used for future work. Another limitation of our study is that stored peripheral blood samples were only available from 81 HCC patients. Therefore, independent validation of these results is required. Given the importance of developing optimal immune specific biomarkers in the “era” of cancer immunotherapy additional studies should confirm or refute these findings.

In summary, pre-operative circulating levels of PD-L1 and Gal-9 do not correlate to their expression in tumor tissue, but have prognostic value in resected HCC patients independently of their expression in tumor tissue. Combined circulating levels and intra-tumoral expression of PD-L1 and Gal-9 provide more confident prognostic immune biomarker profiles compared to each separately.

## Supplementary information

SUPPLEMENTARY INFO

## Data Availability

The datasets used and/or analyzed during the current study are available from the corresponding author on reasonable request.
