# Supplementary material for: Circulating levels of PD-L1 and Galectin-9 are associated with patient survival in surgically treated Hepatocellular Carcinoma independent of their intra-tumoral expression levels
Source: Sci Rep. 2019 Jul 23;9:10677. doi: 10.1038/s41598-019-47235-z (PMC6650499; doi:10.1038/s41598-019-47235-z)
Supplement: Supplementary file 1 — SUPPLEMENTARY INFO [file 41598_2019_47235_MOESM1_ESM.pdf]

Electronic Supplementary Material

Circulating levels of PD-L1 and Galectin-9 are associated with patient survival in surgically treated Hepatocellular Carcinoma independent of their intra-tumoral expression levels

Kostandinos Sideras, Robert A. de Man, Susan M Harrington, Wojciech G. Polak, Guoying Zhou, Hannah M. Schutz, Alexander Pedroza-Gonzalez, Katharina Biermann, Shanta Mancham, Bettina E. Hansen, R. Bart Takkenberg, Anneke J. van Vuuren, Qiuwei Pan, Jan N.M. Ijzermans, Stefan Sleijfer, Dave Sprengers, Haidong Dong, Jaap Kwekkeboom, Marco J. Bruno*

* corresponding author:
Erasmus University Medical Center, Department of Gastroenterology and Hepatology
H-358's, 's Gravendijkwal 230, 3015 CE Rotterdam, The Netherlands
Tel. +31 107035946, Fax. +31 107030352, m.bruno@erasmusmc.nl

Supplementary Table 1: Etiology of liver disease [a]

	N (%)
No known liver disease	20 (24.7)
Alcoholic liver disease	18 (22.2)
Chronic hepatitis-B	14 (17.3)
Chronic hepatitis-C	10 (12.3)
NASH	5 (6.2)
Hemochromatosis	5 (6.2)
Cryptogenic cirrhosis	5 (6.2)
Other [b]	5 (6.2)

[a] When two etiologic factors where present in a single patient only the most dominant etiologic factor was considered, as determined by an experienced hepatologist. Thus the liver disease of three patients with Hepatitis-B sero-positivity was attributed to other concurrent etiologic factors (Hepatitis-C x1, alcoholic liver cirrhosis x1, NASH x1). One patient had both hepatitis C and alcoholic liver cirrhosis as etiology.

[b] Primary biliary cirrhosis x2, primary sclerosing cholangitis x1, autoimmune hepatitis x1, porphyria x1.

Supplementary Table 2:
Multivariate Cox proportional Hazard regression analysis of patients' survival

Variables	HR	95% CI	p-value
TNM classification [a]	1.08	0.35-3.35	.896
Tumor differentiation	1.27	0.59-2.75	.547
Cirrhosis	2.51	0.85-7.41	.095
AFP > 100 μgl^{-1}	5.53	1.62-18.9	**.006**
Combined PD-L1	0.35	0.14-0.83	**.018**
Combined Gal-9	0.18	0.06-0.51	**.001**

[a] American Joint Committee on Cancer. Liver. Amin MB, Edge S, Greene F, Byrd DR, Brookland RK, et al, eds. AJCC Cancer Staging Manual. 8th edition. New York: Springer; 2016.

Supplementary Figure 1

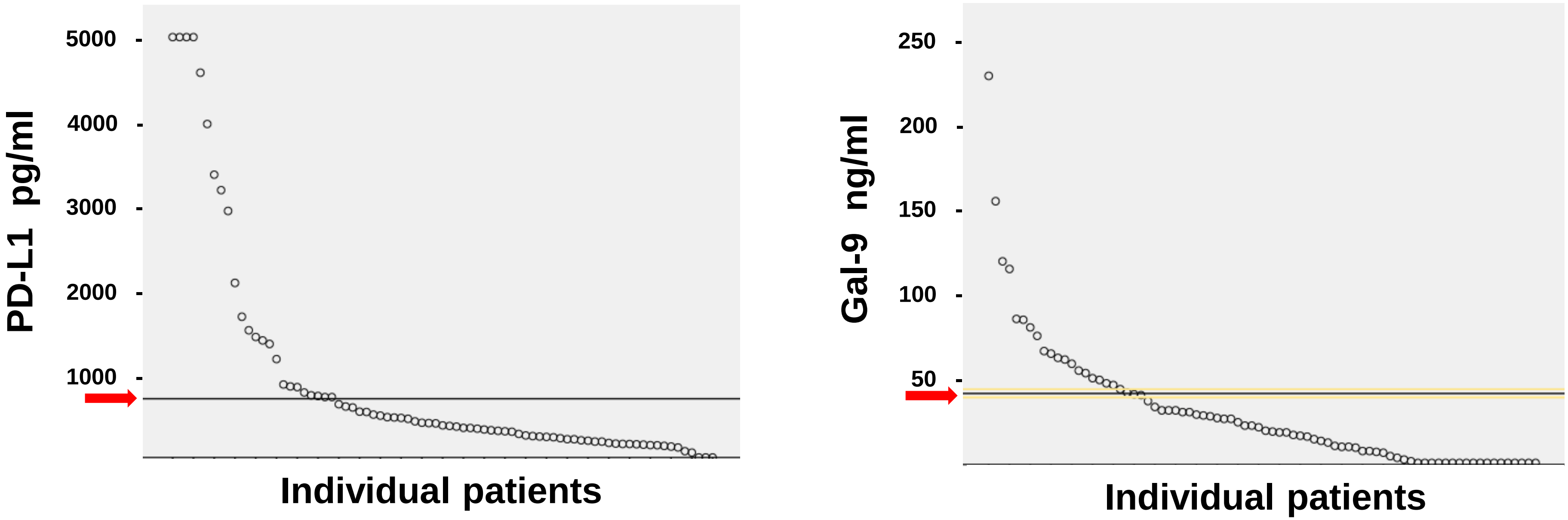

Supplementary figure 1: Plot diagrams of actual PD-L1 and Gal-9 circulating ligand measurement. Red arrows represent the cutoff values used. Note that the PD-L1 Y-axis is terminated at 5000pg/ml.

Supplementary Figure 2

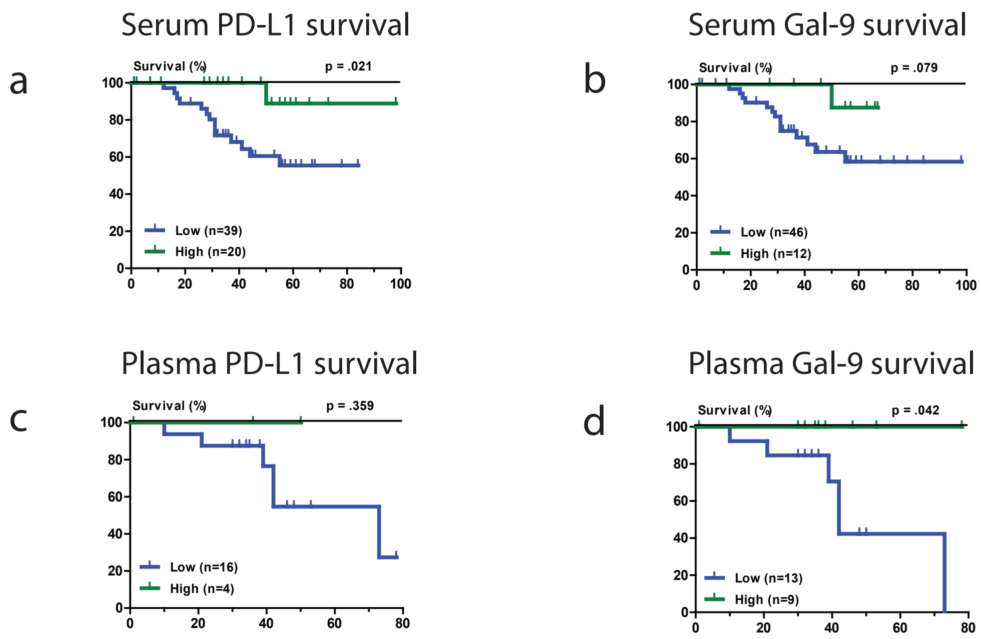

Supplementary figure 2: Kaplan-Meier graphs of circulating PD-L1 and Galectin-9 in patients with serum versus plasma. (a) HCC-specific mortality in relation to serum PD-L1 concentration (n=59). (b) HCC-specific mortality in relation to serum Gal-9 concentration (n=59). (c) HCC-specific mortality in relation to plasma PD-L1 concentration (n=20). (d) HCC-specific mortality in relation to plasma Gal-9 concentration (n=22).

Supplementary Figure 3

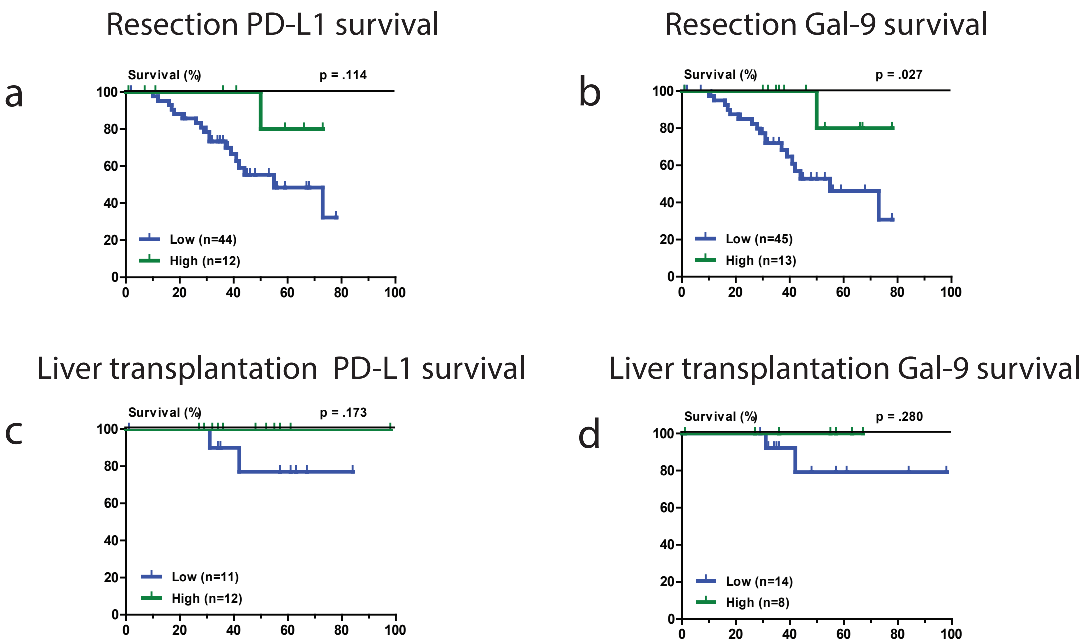

Supplementary figure 3: Kaplan-Meier graphs of circulating PD-L1 and Galectin-9 in patients with resection versus liver transplantation. (a) HCC-specific mortality in relation to PD-L1 concentration of patients with resection (n=58). (b) HCC-specific mortality in relation to Gal-9 concentration of patients with resection (n=58). (c) HCC-specific mortality in relation to PD-L1 concentration of patients with liver transplantation (n=23). (d) HCC-specific mortality in relation to Gal-9 concentration of patients with liver transplantation (n=23).

Supplementary Figure 4

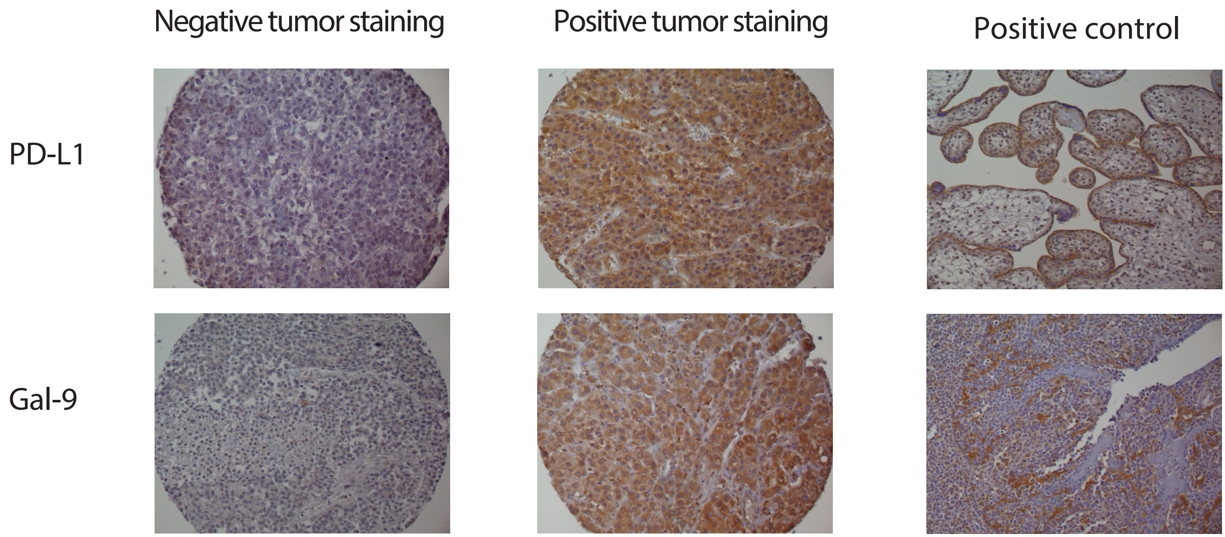

Supplementary Figure 4: Immunohistochemical stainings of PD-L1 and Gal-9 on HCC tumor tissues and positive control tissues (PD-L1: placenta; Gal-9: tonsil).
Images from Sideras et.al., (2017). Oncoimmunology. 2017 Jan 3;6(2):e1273309.

Supplementary Figure 5

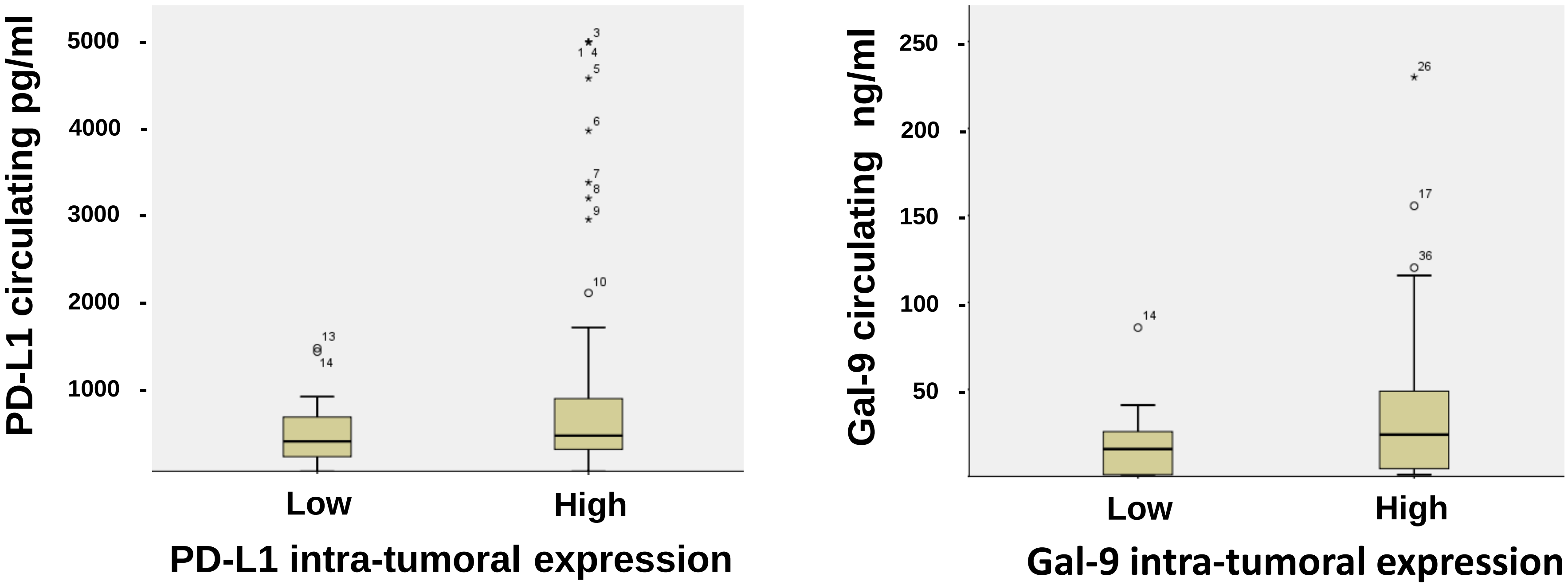

Pearson ChI-Square p=0.42

Pearson ChI-Square p=0.10

Supplementary figure 5: Box-and-whisker plot diagrams of circulating versus intra-tumoral PD-L1 and Gal-9 levels.
